# Knockout of the folate transporter *folt-1 *causes germline and somatic defects in *C. elegans*

**DOI:** 10.1186/1471-213X-10-46

**Published:** 2010-05-04

**Authors:** Misa U Austin, Wei-Siang Liau, Krishnaswamy Balamurugan, Balasubramaniem Ashokkumar, Hamid M Said, Craig W LaMunyon

**Affiliations:** 1 Department of Biological Sciences, California State University Pomona, CA 91768, USA; 2 Current Address: Department of Chemistry and Biochemistry, University of California, Los Angeles, CA 90095, USA; 3 Veterans Affairs Medical Center, Long Beach, CA 90822, USA; 4 Departments of Medicine and Physiology/Biophysics, University of California, Irvine, CA 92697, USA; 5 Current Address: Department of Biotechnology, Alagappa University, Karaikudi 630 003, India

## Abstract

**Background:**

The *C. elegans *gene *folt-1 *is an ortholog of the human reduced folate carrier gene. The FOLT-1 protein has been shown to transport folate and to be involved in uptake of exogenous folate by worms. A knockout mutation of the gene, *folt-1(ok1460)*, was shown to cause sterility, and here we investigate the source of the sterility and the effect of the *folt-1 *knockout on somatic function.

**Results:**

Our results show that *folt-1(ok1460) *knockout hermaphrodites have a substantially reduced germline, generate a small number of functional sperm, and only rarely produce a functional oocyte. We found no evidence of increased apoptosis in the germline of *folt-1 *knockout mutants, suggesting that germline proliferation is defective. While *folt-1 *knockout males are fertile, their rate of spermatogenesis was severely diminished, and the males were very poor maters. The mating defect is likely due to compromised metabolism and/or other somatic functions, as *folt-1 *knockout hermaphrodites displayed a shortened lifespan and elongated defecation intervals.

**Conclusions:**

The FOLT-1 protein function affects both the soma and the germline. *folt-1(ok1460) *hermaphrodites suffer severely diminished lifespan and germline defects that result in sterility. Germline defects associated with folate deficiency appear widespread in animals, being found in humans, mice, fruit flies, and here, nematodes.

## Background

Folate, a member of the B-class of water-soluble vitamins, plays a major role in one-carbon-metabolism that produces nucleotides and several amino acids including methionine [1-3]. Methionine is a substrate for DNA methylation [4], which is an important regulatory mechanism for gene expression during development [5]. Folate is therefore critical to DNA and its expression, but mammals and other multicellular eukaryotes are devoid of the cellular machinery to synthesize folate [6] and must instead rely on active uptake from dietary sources. At the cellular level, three different systems are responsible for folate uptake: the folate receptors [7], the reduced folate carrier (RFC) [8], and the proton coupled folate transporter (PCFT) [9]. The reduced folate carrier is a major folate transport system in mammalian cells and plays an important role in cell growth and development [10].

Folate deficiency, particularly during embryogenesis, can result in a number of developmental defects. In humans, the defects include neural tube deformities [11], anemia [12], cardiovascular abnormalities [13,14], and even cancer [15]. Supplementation with dietary folate during pregnancy is effective in preventing the incidence of neural tube defects by approximately 70% [16,17]. Additionally, genetic variation in the human RFC gene (hRFC) may influence the incidence of folate deficiency defects. Studies have shown that individuals homozygous for a polymorphism (A80G) in hRFC have a slightly higher risk of neural tube defects [18,19] and benefit more from folate supplementation [20,21]. However, a more comprehensive study of six genes involved in folate metabolism found that the risk associated with the A80G polymorphism appears to stem from an interaction with a polymorphism in cystathionine β-synthase [22], a gene involved in the production of cystathionine from homocysteine [23], suggesting a complex set of interactions between RFC and other folate metabolism genes. More universally, it now appears that severe folate deficiency produces embryonic failure in a diversity of species. Mouse embryos die early in development when they are homozygous for an RFC1 knockout allele [24,25], and folate supplementation only delays embryonic death for several days. Females of the fruit fly *Drosophila melanogaster *exposed to the folate analog methotrexate are sterile due to reduced oogenesis and embryonic lethality [26]. Finally, knockout of the folate transporter *folt-1 *in the nematode *Caenorhabditis elegans *induces hermaphrodite sterility [27]. These results suggest a widespread dependency on folate for embryonic development.

We have recently cloned and functionally characterized *folt-1 *from *C. elegans*. This hRFC orthologue transports folate via a specific uptake process that is shared with other folate analogues but not with other water-soluble vitamins (thiamin, biotin and ascorbic acid). Further, transport of folate via the *folt-1 *system is Na^+^-independent, pH-dependent, and DIDS- and sulfasalazine-sensitive. Highest expression of *folt-1 *was found in the pharynx and intestine of adult *C. elegans*, and this uptake system was found to be under both adaptive and developmental regulation. Knocking out of *folt-1 *leads to a significant inhibition in folate uptake with the homozygous mutants being largely sterile (*C. elegans *gene knockout consortium, Oklahoma Medical Research Foundation, Oklahoma City, OK) [27]. The basis of sterility, however, is not known. Also unknown is the effect of this knockout on the nematode soma. We addressed both of these issues in the current study. Our results showed the cause of sterility to be due to defects in germline proliferation, and that the *folt-1 *knockout also leads to diminished *C. elegans *life span and metabolism.

## Results

### *folt-1 *hermaphrodite sterility is due to a germline defect

**Figure 1:**
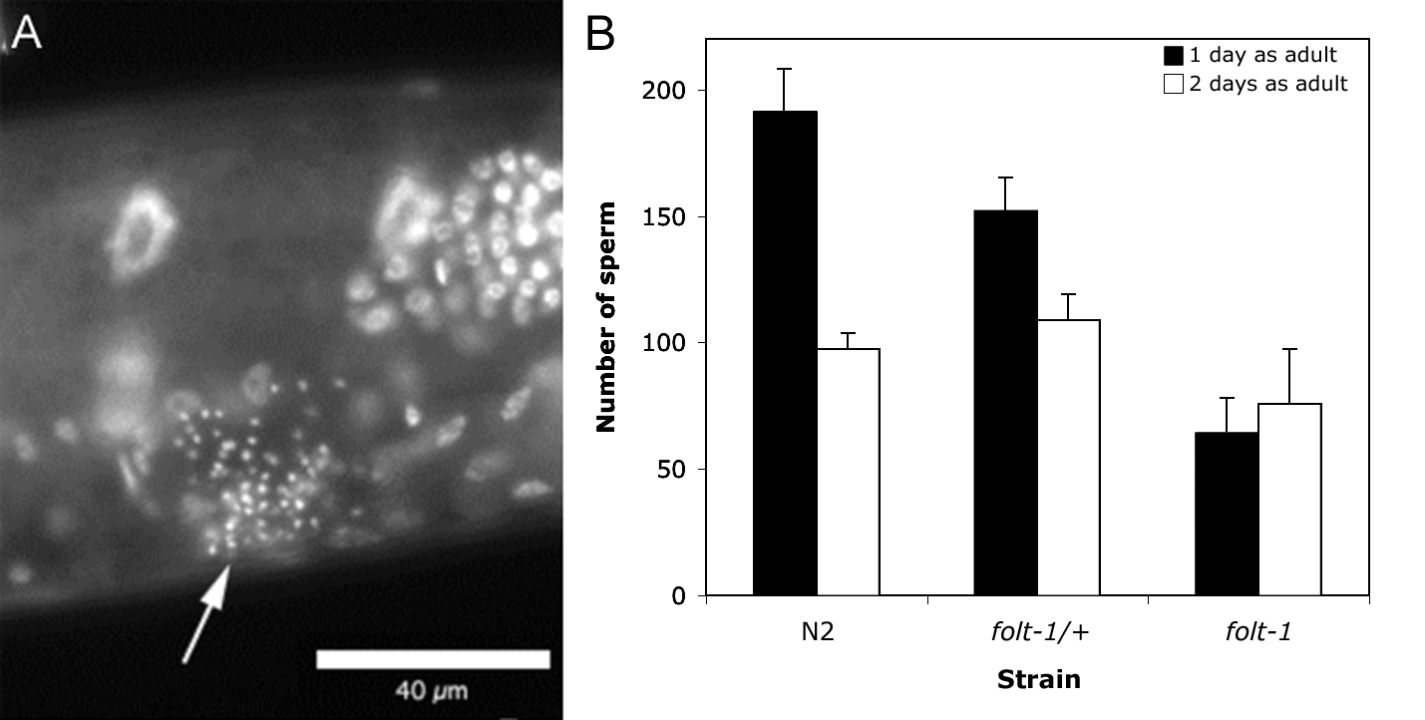
**Sperm production by *folt-1 *knockout hermaphrodites**. (A) The spermathecal region of a *folt-1 *knockout hermaphrodite is shown under epifluorescence after labeling the nuclei with DAPI. The hermaphrodite had been isolated as an L4 larva two days prior. The arrow indicates the compact sperm nuclei that are present in the region of the spermatheca. (B) The number of sperm present within both arms of the reproductive tracts were tallied in homozygous *folt-1 *knockout hermaphrodites (n = 23), *folt-1/+ *heterozygotes (n = 26), and N2 hermaphrodites (n = 30) on the first and second days of adulthood. Error bars represent 1 SEM.

*folt-1 *knockout hermaphrodites are largely sterile. Examined microscopically, we generally found no fertilized eggs, or even unfertilized oocytes, within knockout hermaphrodites. However, nearly a third of knockout hermaphrodites produce a small brood, averaging 7.2 progeny (SEM = 1.1; N = 66), which are themselves sterile. Mutant hermaphrodites produce sperm that were all confined to the spermathecal regions (Fig. 1A), indicating that the knockout hermaphrodites produce motile sperm that can navigate normally. However, *folt-1 *knockout hermaphrodites produced significantly fewer sperm than those produced by either *folt-1/+ *heterozygotes or wild-type hermaphrodites from strain N2 (Fig 1B; *F*_*2,152 *_= 410.87; *P *= 0.001). There was no significant difference between sperm production in *folt-1/+ *heterozygotes and wild-type, indicating that the *folt-1(ok1460) *mutation is recessive for sperm production (Fisher LSD post hoc analysis: *P *= 0.134). Figure 1B shows sperm present in hermaphrodites on the first and second days of adulthood. The number of sperm is smaller on the second day for both *folt-1/+ *heterozygotes and for wild-type worms, reflecting sperm usage for fertilizations. Sperm numbers actually increased on the second day for *folt-1 *knockout mutants, a significant difference compared to the other two strains (strain × day: *F*_*2,152 *_= 7.498; *P *= 0.001). Not only are the sperm unused, they are still being produced well into adulthood, suggesting that gametogenesis proceeds more slowly in *folt-1 *knockout mutants.

**Figure 2:**
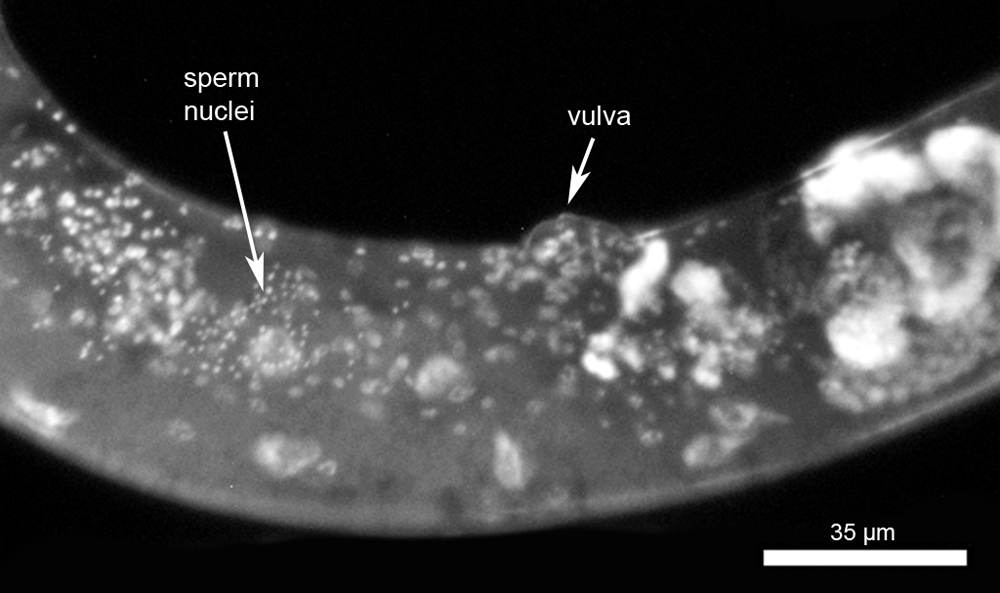
**A mated *folt-1 *hermaphrodite labeled with DAPI**. The arrow indicates location of numerous sperm within the uterus. These sperm were transferred from the mating male(s).

While spermatogenesis is attenuated in *folt-1 *knockout hermaphrodites, oogenesis was nearly absent. The rare oocytes produced by the mutants typically failed to develop into offspring. This failure was not due to a lack of normal sperm, because mating with wild type males did not rescue *folt-1 *hermaphrodite sterility (Table 1). The males in this experiment transferred numerous sperm to the *folt-1 *hermaphrodites, as shown in Fig. 2.

**Table 1:** The effect of mating for *folt-1 *knockout hermaphrodites and males.

Cross progeny production after mating					
Hermaphrodite genotype	Male genotype	Male:Herm. Ratio	Mean no. progeny	SEM	*N*
*folt-1*	N2	4:1	0	0.0	14
*fer-1*	*folt-1; him-14*	4:1	65.9	27.0	11
*fer-1*	*folt-1; him-14*	1:10	15.9	10.9	15
*fer-1*	*him-14*	1:10	49.8	12.6	15

The *him-14(it144)II *mutation was used to obtain males with the *folt-1(ok1460) *knockout mutation. Unmated hermaphrodites that are *him-14 *mutants produce male progeny [59].

**Figure 3:**
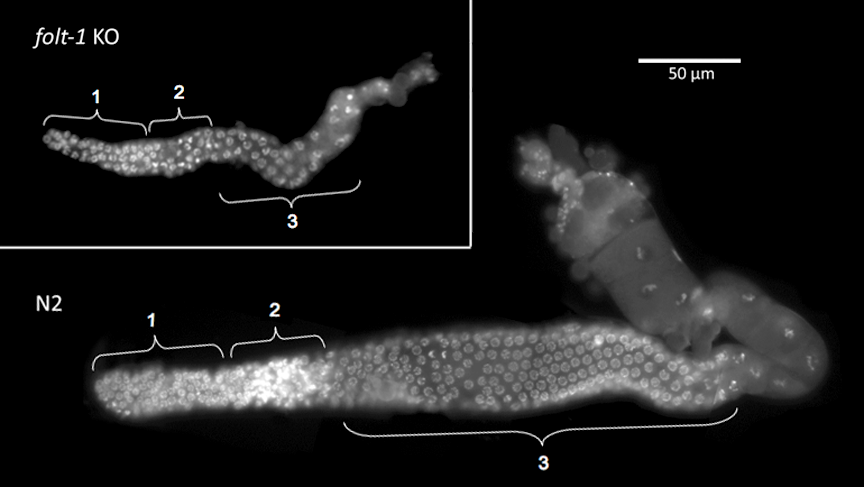
**Germ nuclei in the reproductive tracts of hermaphrodites**. The N2 reproductive tract on the bottom shows nuclei in the mitotic phase (1), a transitional phase (2), and the pachytene phase (3). The *folt-1 *knockout reproductive tract on the top is much smaller, but nuclei in the three phases are present. The nuclei were visualized under epifluorescence after DAPI labeling.

**Figure 4:**
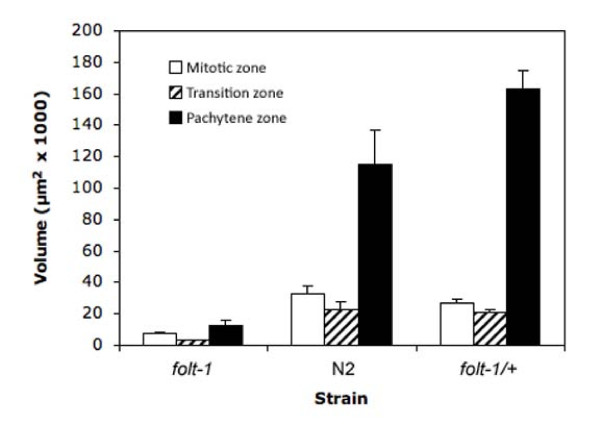
**Volumes of the three zones of the reproductive tracts of *folt-1 *knockout mutants, N2, and *folt-1/*+ heterozygote hermaphrodites**. Error bars represent 1 SEM.

The *C. elegans *hermaphrodite reproductive tract has two independent arms, each containing a region of densely packed syncytial nuclei. The nuclei in the most distal zone are mitotic, while those in the more proximal zone have entered the meiotic pachytene stage with a transitional zone in between (Fig. 3) [28]. The volumes of the three zones were significantly different among the strains examined (Fig. 4; Mitotic: *F*_*2,24 *_= 53.4; *P *< 0.001; Transition: *F*_*2,24 *_= 44.6; *P *< 0.001; Pachytene: *F*_*2,24 *_= 86.9; *P *< 0.01). All three zones of the *folt-1 *knockout mutants were significantly smaller than those of either N2 or the *folt-1/+ *heterozygotes at *P *= 0.001 by Bonferroni post hoc tests. N2 and *folt-1/+ *heterozygotes differed significantly only in the volume of the Pachytene zone; *folt-1/+ *heterozygotes had a larger Pachytene zone (*P *= 0.001, Bonferroni post hoc test).

The *folt-1 *knockout therefore reduces the presence of germ nuclei. This reduction could be due to a slower rate of nuclear division. Alternatively, the reduction could be due to a greater rate of apoptosis, which occurs normally in the *C. elegans *hermaphrodite reproductive tract [29]. To distinguish these two possibilities, we examined apoptosis in the hermaphrodite reproductive tract. We determined the number of nuclei undergoing apoptosis using dyes that label apoptotic nuclei in the reproductive tract [29]. Both N2 (n = 16) and *folt-1/*+ heterozygotes (n = 22) had on average 1.5 apoptotic nuclei, but *folt-1 *knockout mutants (n = 30) contained significantly fewer apoptotic nuclei, averaging 0.1 (*F*_*2,65 *_= 17.06; *P *< 0.001). Therefore, *folt-1 *knockout mutants appear to have a defect in the germline proliferation that populates the reproductive tract with germ nuclei.

### *folt-1 *knockout males are fertile but suffer germline and mating defects

*folt-1 *knockout males are capable of siring progeny, but they are not as fertile as wild-type males. We tested the ability of knockout males to sire progeny in two mating densities: (i) four knockout males to one *fer-1(hc13ts) *hermaphrodite, and (ii) one knockout male to 10 *fer-1(hc13ts) *hermaphrodites for two days in both experiments (Table 1; the *fer-1(hc13ts) *mutation incapacitates sperm when worms are reared at 25°C, but *fer-1 *mutant hermaphrodites will become fertile if mated with fertile males [30]). In both experiments the knockout males sired progeny: nearly 70 progeny when four *folt-1; him-14 *knockout males mated with a single *fer-1 *hermaphrodite mated, but only ca. 15 offspring when one knockout male was paired with 10 hermaphrodites (Table 1). These results clearly show that the *folt-1(ok1460) *mutation does not prevent the production of functional sperm in males. However, control males paired with 10 *fer-1 *hermaphrodites sired nearly four times as many progeny compared to the knockout males (Table 1).

**Figure 5:**
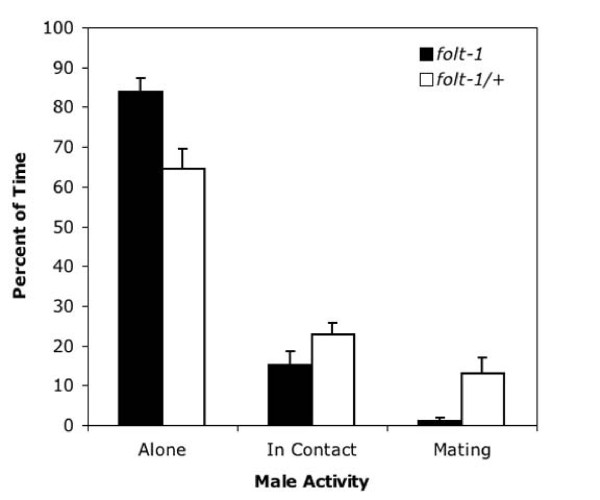
**The mating efficiency of *folt-1 *knockout males compared to *folt-1/+ *heterozygous males**. The activity of single males confined with 10 wild type hermaphrodites was observed at five minute intervals for an hour. Homozygous *folt-1 *knockout males were observed to be alone a greater percentage of our observations, and to be mating much less than were *folt-1/+ *heterozygotes.

**Figure 6:**
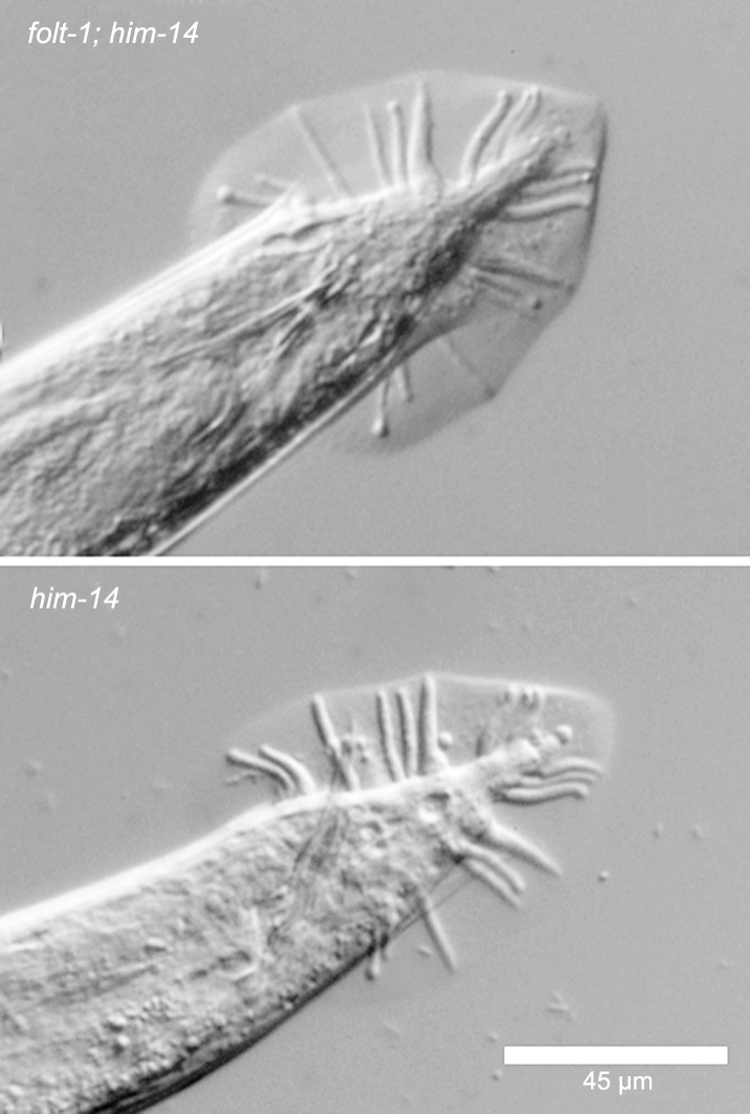
**The copulatory bursae from the tails of both folt-1; him-14, and him-14 males**. There was no observable difference between the tails of the two genotypes.

**Figure 7:**
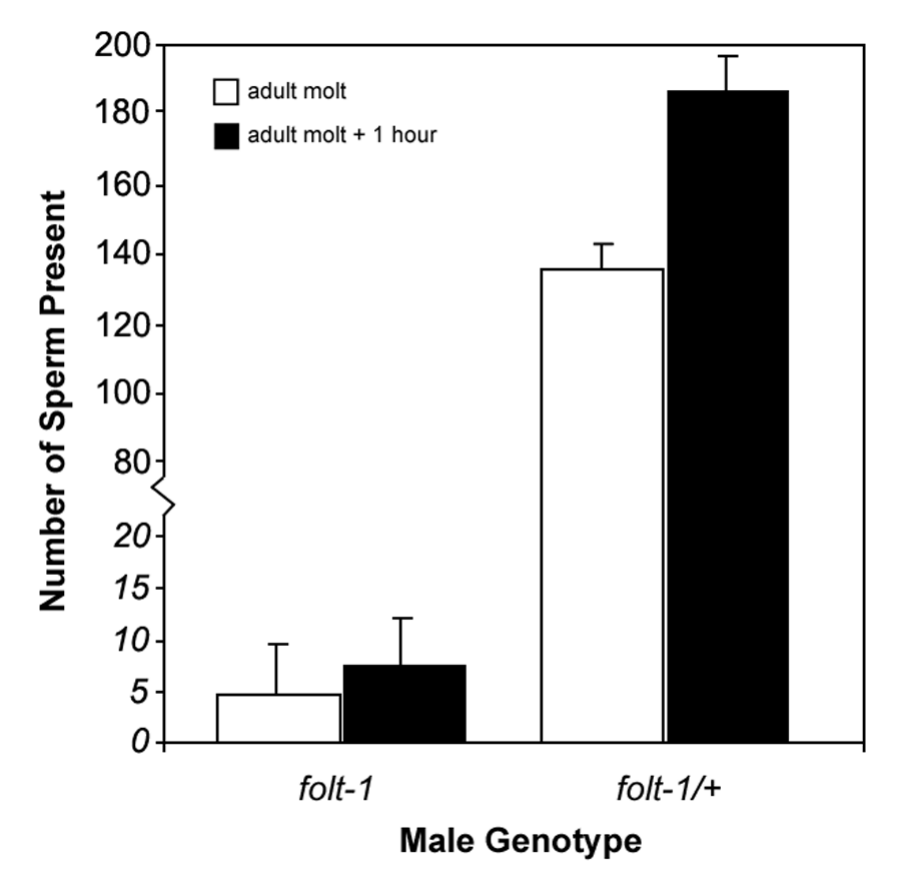
**Sperm production by *folt-1 *knockout mutant and *folt-1/+ *heterozygous males**. Sperm numbers within the reproductive tracts were tallied at the adult molt and one hour later. *folt-1*/+ heterozygous males produced wild-type numbers of sperm, but *folt-1 *knockout mutants had very few sperm, producing only about 3 per hour.

We investigated the reduced cross progeny sired by *folt-1 *knockout males. First, we assayed mating efficiency. Knockout males spend significantly less time *in copula *than do males heterozygous for the *folt-1 *knockout mutation (Fig. 5; *t *= 2.06, *P *= 0.003). Because mating efficiency is severely impacted by defects in the male tail [31], we investigated tail morphology. Tail morphology is not the cause of reduced male fertility, because the tails of knockout males (n = 16) appeared identical to those of control males (n = 14; Fig. 6). Finally, we determined the rate of sperm production in knockout males. Wild-type males that have been adult for one day are known to produce sperm at a rate of nearly 60 per hour [32]. We tallied the numbers of sperm within the reproductive tracts of *folt-1 *knockout males and *folt-1/+ *heterozygote males at two ages: the adult molt and one hour past the adult molt (Fig. 7). Comparing the sperm numbers at the two time points indicates that the *folt-1/+ *heterozygote males produced wild type numbers of sperm, averaging 51 per hour, but homozygous *folt-1 *knockout males produced only 3 sperm per hour, a difference that was significant (strain × age interaction: *F*_*1,33 *_= 5.56, *P *= 0.024).

### *folt-1 *worms have reduced longevity and metabolism

**Figure 8:**
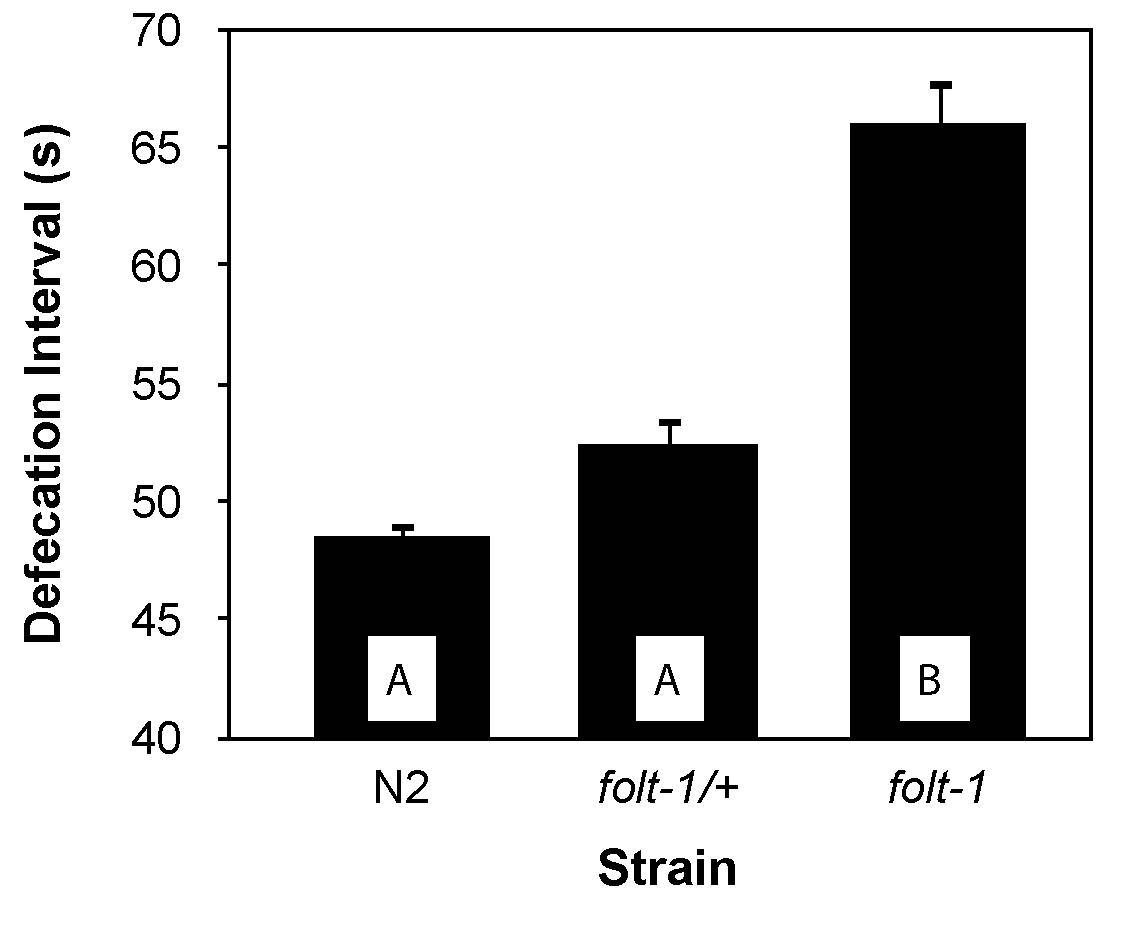
**The time interval between defecations for *folt-1 *knockout worms, *folt-1/+ *heterozyogotes, and wild type worms from the strain N2**. Error bars represent 1 SEM, and bars sharing letters are not significantly different at *P *= 0.05 by Bonferroni post hoc analysis.

**Figure 9:**
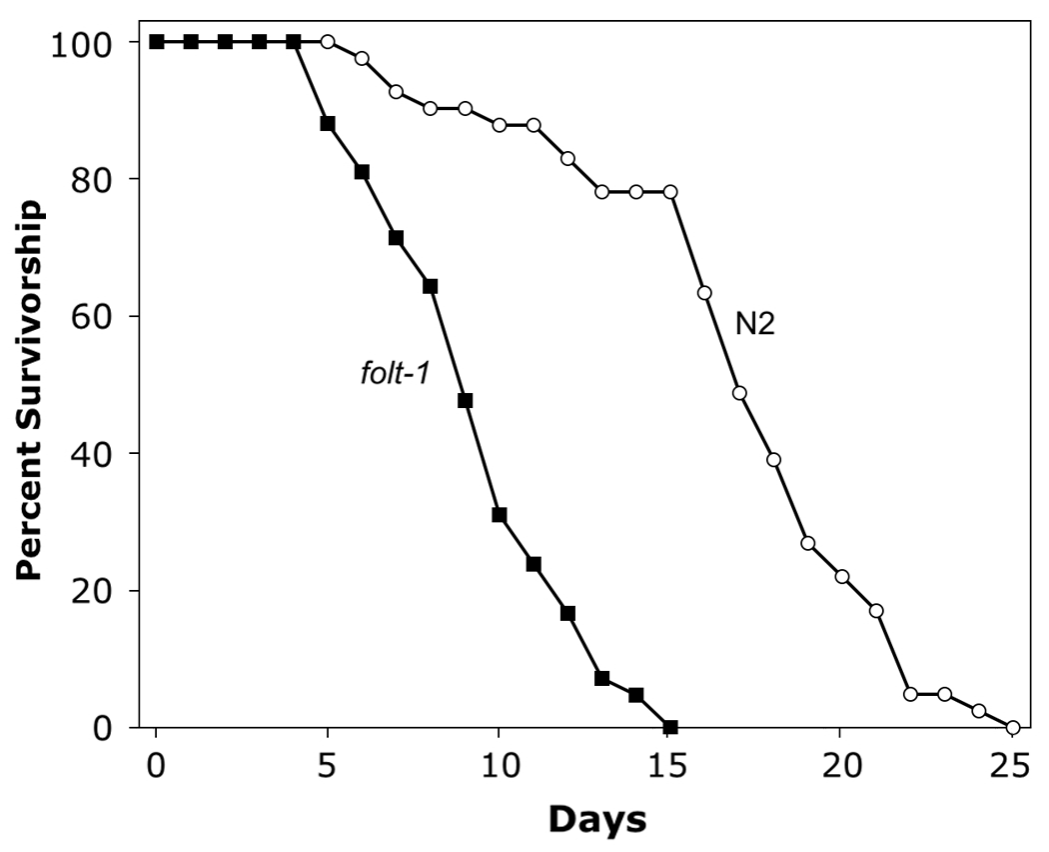
**The survivorship of *folt-1 *knockout mutant worms compared to that of N2 worms**.

Because *folt-1 *worms lay very few eggs and have reduced production of germ nuclei, we wondered if they might be compromised metabolically. We first measured the rate of defecation, which is sensitive to variations in energy metabolism [33]. *folt-1 *knockout worms defecated at a significantly reduced rate compared to *folt-1/+ *heterozygotes and to wild type (*F*_*2,33 *_= 44.48, *P *< 0.001; Fig. 8). Because defecation rates suggested that *folt-1 *knockout mutants experienced reduced metabolism, we wondered if they might have extended lifespans [34,35]. Our lifespan results ran counter to the hypothesis: *folt-1 *knockout worms had significantly shorter life spans of 9.4 ± 0.4 (SEM) days compared to N2 worms which lived 16.9 ± 0.7 days (*t *= 9.07; *P *< 0.001; Fig. 9). The decrease in average life span due to the *folt-1 *knockout was 45%. Therefore, in addition to its effects on the germline, our lifespan and defecation results indicate that the *folt-1 *gene plays a role in the soma.

## Discussion

The *folt-1(ok1460) *knockout mutation affects both the soma and the germline. Mutant hermaphrodites defecate slower, live significantly shorter lives, have reduced germline proliferation, produce small numbers of sperm and are oogenesis defective. These findings suggest that *folt-1 *is expressed in multiple tissues. Using a transcriptional *folt-1::GFP *fusion, Balamurugan *et al *[27] demonstrated that *folt-1 *is expressed primarily in the posterior intestine and pharynx of the digestive system but also in numerous muscles. The germline defects we describe here implicate the gonadal tissue as an additional site of *folt-1 *expression, but microarray data indicate that *folt-1 *is not upregulated in the germline [36]. In a large-scale analysis of gene expression in *C. elegans *using GFP transcriptional fusions, Hunt-Newbury *et al*. [37] showed that *folt-1 *is expressed in the gonadal sheath cells. These muscular cells are known to move the oocytes out of the ovary and into the sperm storage chamber during ovulation [38], but they also form gap junctions with the oocytes [39] providing a possible route for folate into the developing germ cells. It is interesting that the reproductive tracts of *folt-1 *knockout hermaphrodites appear remarkably similar to those that develop after the larval ablation of the gonadal sheath/spermathecal precursor cells as shown by McCarter *et al*. [40]. Perhaps the germline proliferation defect induced by ablation of the sheath cell lineage is due to its effect on folate transport into the germline.

While the gonadal sheath cells may be the conduit into the germline, it is unclear how the folate deficit associated with the *folt-1(ok1460) *knockout results in defective proliferation. Germline proliferation is controlled by cells at the distal tips of the gonad arms. These distal tip cells maintain the mitotic stem cell population of nuclei through a Notch family signaling pathway [41]. At this point, it is unclear whether folate is affecting Notch signaling from the distal tip cells to the mitotic cells in *C. elegans*, but in vertebrates, low folate levels are known to modulate intracellular signaling [42]. In mice, knockout of the orthologous gene (*Slc19a1*) results in sterility due to embryonic failure at a very early stage [24,25]. Taparia *et al*. [25] hypothesize that folate deficiency in mice causes a buildup of homocysteine which in turn causes an immune response to folate receptor 1 (*Folr1*). In support of this hypothesis, potentially folate deficient women who had pregnancies complicated by neural tube defects tended to have autoantibodies toward their folate receptors [43]. Such a mechanism could not occur in *C. elegans *because they have neither a folate receptor ortholog [27], nor a mammalian immune system. Alternatively, folate affects DNA methylation [4], and DNA methylation is an important form of gene regulation in the soma of vertebrate embryos [5]. DNA methylation cannot explain our results, because it is not found in *C. elegans *and other nematodes [44]. The remaining known scenarios by which the *folt-1(ok1460) *knockout could induce sterility involve the production of nucleotides and amino acids [1-3]. Deficits in DNA synthesis and/or gene expression would likely be manifest in the gonad, the only site of cell division in the adult worm, and proliferation of germ nuclei is significantly reduced in *folt-1 *knockout worms.

*C. elegans *has been extensively used as an animal model for studying the effect of different factors/conditions on aging [45] and metabolism [46,42,47]. In this study we found that folate deficiency induced by the *folt-1 *knockout reduces lifespan and slows defecation, a behavior sensitive to metabolic rate [33]. Furthermore, knockout males are poor maters, even though their copulatory organs appear normal, likely reflecting a slowed metabolism and/or additional somatic defects. These effects may also be due to the critical role of folate in the biosynthesis of purines, pyrimidines, and certain amino acids. However, comparing the *folt-1 *knockout phenotype to those of other mutants suggests alternative explanations. Many mutations that slow metabolism tend to extend lifespan [48,49], but a few metabolic mutations reduce longevity [50] as does the *folt-1(ok1460) *mutation. For example, longevity is reduced by a mutation in *mev-1*, which encodes a subunit of *cytochrome b*_*560 *_[51,52], and mutant worms are thought to have higher levels of reactive oxygen species. In addition, the *uaDf5 *mitochondrial deletion reduces both metabolic rate and lifespan [53], and we have shown that worms harboring the *uaDf5 *deletion have increased ROS (CWL, unpublished). Perhaps *folt-1 *knockout worms have increased ROS as well, although this issue awaits further study.

## Conclusions

Our results suggest that folate deficiency in *C. elegans *caused by the *folt-1(ok1460) *mutation produces an overall decline in worm fitness and a defect in germline proliferation. Folate deficiency produces defects in oogenesis in humans [54], mice [55], the fruit fly *D. melanogaster *[26]. We show here that folate deficiency reduces production of sperm and nearly eliminates oogenesis in the nematode *C. elegans*. While the specific mechanisms for the defects remain unclear, there appears to be a general requirement for folate during oogenesis in animals.

## Methods

### Worm handling

Worm strains were maintained on Nematode Growth Media (NGM) agar plates seeded with *Escherichia coli *bacteria OP50 [56]. Experiments were conducted at 20°C unless noted otherwise. The *C. elegans *strain N2 was used as the wild-type control. The *him-14(it144)II *and *folt-1(ok1460)V/nT1 [qIs51](IV;V) *strains were obtained from the *Caenorhabditis *Genetics Center (CGC, Minneapolis, MN). Unmated *him-14 *mutant hermaphrodites produce a relatively high proportion of male progeny. The *folt-1(ok1460)V/nT1 [qls51](IV;V) *strain was originally provided by the *C. elegans *Reverse Genetics Core Facility at UBC, which is part of the International *C. elegans *Gene Knockout Consortium (Oklahoma Medical Research Foundation, Oklahoma City, OK). This strain bears the *ok1460 *deletion allele balanced by the *nT1 *translocation that is itself marked with the *qls51 *GFP transgene under the *myo-2 *promoter, which induces pharyngeal expression. The strain was maintained by picking GFP-labeled worms, which are themselves heterozygotes for the *ok1460 *mutation. Knockout mutants were obtained by picking worms without pharyngeal GFP. Knockout males were obtained in one of two ways. First, we picked the rare males produced through non-disjunction of the X chromosome and mated them to hermaphrodites to establish a male-producing line of *folt-1(ok1460)V/nT1 [qls51](IV;V)*, which had to be propagated by setting up matings every generation. Second, we constructed the strain *him-14(it144)II; folt-1(ok1460)V/nT1 [qls51](IV;V)*, which generates males from unmated hermaphrodites through increased rates of non-disjunction. The *fer-1(hc13ts) *was obtained from Samuel Ward at University of Arizona; *fer-1(hc13ts) *mutant hermaphrodites are sterile at 25°C due to a sperm defect.

### Microscopy for Sperm Counts, Reproductive Tract Morphometry, and Germline Apoptosis

Worms were observed under DIC optics for structural examination, especially inspection of the tail morphology of *folt-1 *knockout males. To count sperm within worms, we fixed them, labeled their DNA with DAPI (4'6-diamidino-2-phenylindole), and observed them under epifluorescence [53]. DAPI labeled sperm nuclei are characteristically compact in nature, making them easy to identify and count. This technique was used to count sperm produced by *folt-1 *knockout hermaphrodites and males, and it was used to determine whether *folt-1 *knockout hermaphrodites had received sperm from males at mating. DNA labeling was also used in a morphometric analysis of the reproductive tract of mutant worms. One day after they were picked as L4 larvae, knockout mutant hermaphrodites and control worms were dissected under Sperm Medium [57] containing 10 μg/ml Hoechst 33342, a DNA label similar to DAPI, but able to penetrate living tissue. The reproductive tracts were imaged and the nuclear characteristics used to define the mitotic, pachytene, and transitional regions of the tracts. The regions were measured using OpenLab™ imaging software, and their cylindrical volumes calculated. Finally, we examined the hermaphrodite germline for apoptosis using an assay involving the dye SYTO12 (Molecular Probes^®^) as described by Gumienny *et al*. [29]. Briefly, one day after isolation as L4 larvae, adult hermaphrodites were exposed for 4-5 hours to a 33 μM aqueous solution mix of SYTO12 and 10 μg/ml Hoechst 33342. Subsequently, these animals were transferred to seeded plates for 30-60 minutes to remove stained bacteria from their guts. Lastly, the worms were mounted on agarose pads and examined under epifluorescence. Apoptotic nuclei are labeled by SYTO12.

### Lifespan and Defecation

Worm lifespan was measured with age-synchronous animals from the time they were eggs until their death. To obtain age-synchronized cohorts of worms, adult hermaphrodites were allowed to lay eggs for two hours in 35 mm Petri dishes. The resulting eggs were allowed to hatch and the worms grown at 20°C. During their fertile period, the worms were transferred to new plates daily in order to distinguish them from their progeny. Worms were examined every day until death and were scored as dead when they were no longer able to move, even in response to touch. Defecation interval was determined by observing worms for the obvious posterior body contractions that load the rectum for defecation [58]. We measured three defecation intervals for each worm and took the mean.

### Statistical Analyses

All statistical analyses were conducted using PASW Statistics 17.0. Pairs of means were analyzed with *t*-Tests, whereas data sets with 3 or more means were analyzed by ANOVA, with post-hoc comparison of pairs of means with either Bonferroni or Fisher's LSD tests.

## Authors' contributions

MUA and WSL contributed to experimental design, data collection, and manuscript preparation. KB and BA participated in the conception and design of the projects as well as manuscript preparation. HMS contributed to experimental design, data interpretation, and manuscript preparation. Finally, CWL took part in designing and conducting the experiments, analyzing the data, and preparing the manuscript. All authors read and approved the final manuscript.
